# Specific Human Milk Oligosaccharides Differentially Promote Th1 and Regulatory Responses in a CpG-Activated Epithelial/Immune Cell Coculture

**DOI:** 10.3390/biom13020263

**Published:** 2023-01-31

**Authors:** Marit Zuurveld, Veronica Ayechu-Muruzabal, Gert Folkerts, Johan Garssen, Belinda van‘t Land, Linette E. M. Willemsen

**Affiliations:** 1 Division of Pharmacology, Utrecht Institute for Pharmaceutical Sciences, Faculty of Science, Utrecht University, 3584 CG Utrecht, The Netherlands; 2 Danone Nutricia Research B.V., 3584 CT Utrecht, The Netherlands; 3 Center for Translational Immunology, University Medical Center Utrecht, 3584 CX Utrecht, The Netherlands

**Keywords:** human milk oligosaccharides, intestinal epithelial cells, mucosal immunity, non-digestible oligosaccharides, early life nutrition

## Abstract

Proper early life immune development creates a basis for a healthy and resilient immune system, which balances immune tolerance and activation. Deviations in neonatal immune maturation can have life-long effects, such as development of allergic diseases. Evidence suggests that human milk oligosaccharides (HMOS) possess immunomodulatory properties essential for neonatal immune maturation. To understand the immunomodulatory properties of enzymatic or bacterial produced HMOS, the effects of five HMOS (2′FL, 3FL, 3′SL, 6′SL and LNnT), present in human milk have been studied. A PBMC immune model, the IEC barrier model and IEC/PBMC transwell coculture models were used, representing critical steps in mucosal immune development. HMOS were applied to IEC cocultured with activated PBMC. In the presence of CpG, 2′FL and 3FL enhanced IFNγ (*p* < 0.01), IL10 (*p* < 0.0001) and galectin-9 (*p* < 0.001) secretion when added to IEC; 2′FL and 3FL decreased Th2 cell development while 3FL enhanced Treg polarization (*p* < 0.05). IEC were required for this 3FL mediated Treg polarization, which was not explained by epithelial-derived galectin-9, TGFβ nor retinoic acid secretion. The most pronounced immunomodulatory effects, linking to enhanced type 1 and regulatory mediator secretion, were observed for 2′FL and 3FL. Future studies are needed to further understand the complex interplay between HMO and early life mucosal immune development.

## 1. Introduction

The third most abundant solid component in human milk are the human milk oligosaccharides (HMOS). HMOS are highly variable and complex fibers with over 150 different structures identified [1]. Each mother possesses a unique combination of HMOS depending on genetics, diet, environment and stage of lactation [2,3]. Neonates are unable to digest HMOS, but these can be fermented by the intestinal microbiome [4]. However, HMOS can also cross the mucosal linings since they can be detected in the serum and urine of breastfed infants [5,6,7]. More evidence is gathering that HMOS possess immunomodulatory properties important for neonatal immune maturation via the intestinal microbiome and/or by directly affecting both mucosal and systemic immunity [8]. Deviations in neonatal immune maturation may have life-long effects, such as predisposing the infant to develop immune related disorders, such as allergic diseases [9]. 

The presence of HMOS is limited in commercial formula feeding; however, infant formulas are mostly supplemented with prebiotic non-digestible oligosaccharides such as long chain fructo-oligosaccharides (lcFOS) and short chain galacto-oligosaccharides (scGOS). These prebiotic fibers can suppress the production of pro-inflammatory cytokines and promote the diversity of the microbiome, similar but not identical to breastfed infants [10,11]. Recent developments in large-scale chemical production of HMOS have presented the potential of supplementing formula with several types of manufactured HMOS [12], potentially further improving immune development in formula fed infants. Several HMOS can be manufactured by bacterial production, such as genetically modified bacteria [13,14,15,16] as well as enzymatic conversion of lactose [17,18]. Although the final products should be similar, contaminants, such as endotoxins, may influence immunological responses to these products.

2′-Fucosyllactose (2′FL) is the most abundant HMOS present in human milk; however, not all mothers express this HMOS and produce 3-fucosyllactose (3FL) instead. This is due to a variation in the FUT2 (fucosyltransferase 2) gene that adds a fucose group via α1-2 linkage onto galactose [19]. In addition, the secretion of 2′FL decreases during lactation while 3FL secretion increases over time [20]. Therefore, it would be of interest to investigate whether 3FL has similar or complementary immunomodulatory properties compared to 2′FL. Despite the structural resemblance, the binding of the fucose group to glucose, as opposed to galactose, may result in differential immunological outcomes as binding affinity to immune receptors is affected by these structural differences [21,22]. In addition, similar differences are observed in the structurally comparable sialylated HMOS 3′-sialyllactose (3′SL) and 6′-sialyllactose (6′SL). The secretion of 3′SL in human milk increases over time while 6′SL concentrations are found to decline [20]. Although a large proportion of the HMOS contain a fucose or sialic acid group, many of the HMOS structures are composed without these terminating groups; lacto-*N*-neotetraose (LNnT) is an example of such undecorated HMOS [1]. 

The early life induction of tolerance and strengthening of Th1 type immunity is of importance due to the infant’s Th2-skewed immune system, which may pose the neonate at risk to develop allergies [23]. A dominant presence of Th2 type cytokines, such as IL13, disturbs intestinal epithelial integrity, increasing the leakage of allergens into the lamina propria [24]. Maintaining barrier integrity and stimulation of tolerogenic signals induces the regulation of immune responses through the tolerogenic polarization of the intestinal epithelial cells (IEC) and crosstalk with underlying immune cells. Tolerogenic signals result in the release of TGFβ, galectin-9 and retinoic acid by IECs, which promotes the development of regulatory T (Treg) cells, homeostasis and protection of mucosal surfaces [25,26].

A specific mixture of non-digestible oligosaccharides (9:1 mixture of scGOS and lcFOS) co-incubated with synthetic CpG, a TLR9 ligand used to mimic the presence of intestinal bacterial DNA, increased the secretion of galectin-9 from IEC and subsequently promoted the release of IFNγ and IL10 by peripheral blood mononuclear cells (PBMCs) in an in vitro IEC/PBMC coculture model [27]. Next to the scGOS/lcFOS mixture, 2′FL promotes Th1 type and regulatory immune development as well by increasing galectin-9 and TGFβ release from the intestinal epithelium upon exposure to the oligosaccharides and CpG [28]. In addition, a coculture of IEC exposed to non-digestible oligosaccharides and CpG with activated PBMCs resulted in enhanced IFNγ and IL10 and reduced IL13 secretion [28].

**Figure 1:**
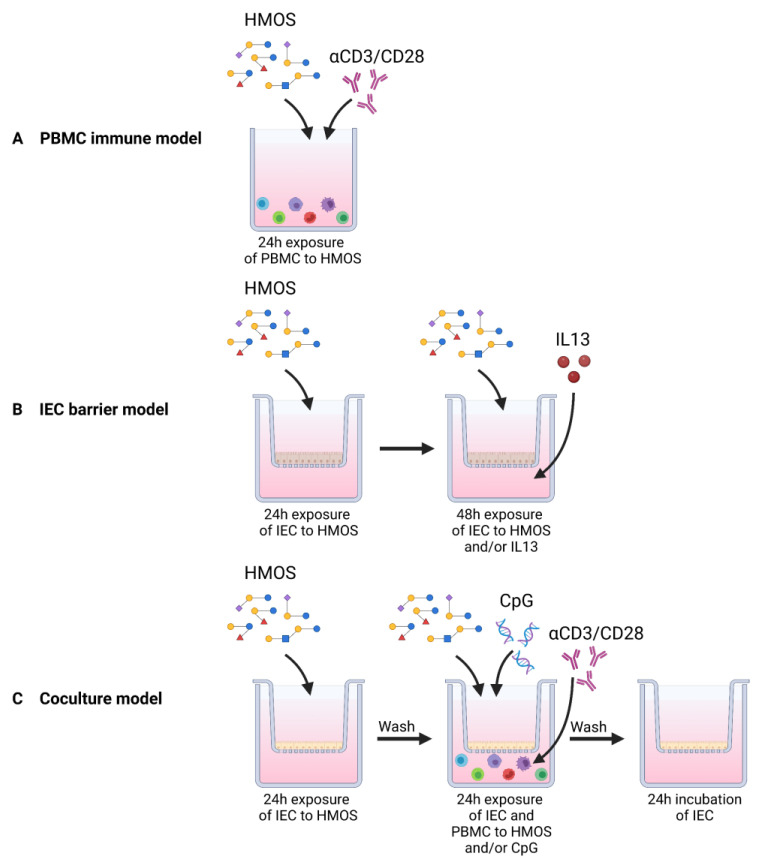
Schematic overview of the different models described in this manuscript. (**A**) The PBMC immune model allowed for direct exposure of activated PBMCs to HMOS. Freshly isolated PBMC were activated using anti-CD3 and anti-CD28, while being exposed to HMOS for 24 h. (**B**) In the IEC barrier model, T84 cells were cultured in transwell inserts for 4 weeks. Cells were apically pre-exposed to HMOS for 24 h prior to basolateral addition of IL13 to induce barrier disruption. (**C**) For the IEC/PBMC coculture model, human intestinal IEC (HT29 cells) were cultured on transwell inserts until confluency was reached. IEC were apically exposed to HMOS for 24 h. After preincubation with HMOS, the apical an basolateral medium was refreshed. αCD3/CD28-activated PBMCs were added to basolateral compartment. IEC were apically exposed to HMOS again either in presence or absence of CpG. After 24 h of coculture, basolateral supernatant was collected to measure cytokine secretion and PBMCs were collected for phenotypical analysis. IEC were again washed and incubated for another 24 h in fresh medium to measure epithelial mediator release into the basolateral compartment.

In this current manuscript, we studied the immunomodulatory effects of five enzymatically produced HMOS (see Table 1), which are present in human milk, using three different in vitro models for the intestinal barrier and (mucosal) immune functioning (Figure 1). In addition, the immunomodulatory effects of enzymatically produced 2′FL and 3FL were compared to bacterial produced 2′FL and 3FL to study the impact of production method on the immunomodulatory effects. Finally, the role of IEC in the immunomodulatory effects of the HMOS was investigated. The secretion of cytokines from both IEC and PBMCs and phenotype of PBMCs was determined to assess functional immune outcomes.

## 2. Materials and Methods

### 2.1. Isolation of Human Peripheral Blood Mononuclear Cells

Human PBMCs were purified from buffy coats from healthy donors (Sanquin, Amsterdam, The Netherlands). Cells were separated by density gradient centrifugation (1000× *g*, 13 min) and washed with PBS containing 2% FCS. An enriched cells fraction was harvested, and erythrocytes were lysed for 5 min using a red blood cell lysis buffer (4.14 g NH_4_CL, 0.5 g KHCO_3_, 18.6 mg Na_2_EDTA in 500 mL demi water, sterile filtered, pH = 7.14). PBMCs were carefully resuspended in RPMI1640 (Gibco, Waltham, MA, USA) supplemented with 2.5% FCS, penicillin (100 U/mL) and streptomycin (100 μg/mL). 

### 2.2. Preparation of HMOS

Lyophilized enzymatically (lactose-derived) produced 2′FL, 3FL LNnT, 3′SL and 6′SL (Carbosynth, Berkshire, UK) and bacterially (*E. coli*) produced 2′FL and 3FL (Jennewein Biotechnologie GmbH, Rheinbreitbach, Germany) (Table 1) were dissolved in plain McCoy’s 5A or DMEM/F12 medium (Gibco), consequently sterile filtered (0.2 μm filter) and stored at −20 °C until further use. 

### 2.3. PBMC Immune Model

Freshly isolated PBMCs (2 × 10^6^ cells/mL) were activated with anti-CD3 (150 ng/mL, clone CLB-T3/2) and anti-CD28 (100 ng/mL, clone CLB-C28) (Sanquin, Amsterdam, The Netherlands). A fraction (estimated to be up to 4%) of the ingested HMOS could be transported over the intestinal epithelium and become systemically available [6,7,29]. Therefore PBMCs were directly exposed to 0.01%, 0.05% or 0.1% (*w*/*v*) concentrations of HMOS for 24 h to mimic the systemic availability of low HMOS concentrations. After 24 h, viability of the PBMCs was assessed, and supernatants were collected and stored at −20 °C to determine cytokine levels. 

### 2.4. IEC Barrier Model

T84 cells were grown in 75 cm^2^ flasks until 80% confluency in DMEM/F12 medium (Gibco, Waltham, MA, USA) supplemented with Glutamax (Invitrogen, Waltham, MA, USA), 10% fetal calf serum (FCS) and the antibiotics penicillin (100 U/mL) and streptomycin (100 μg/mL) (Sigma-Aldrich, Saint Louis, MO, USA). After trypsinization, samples were 5 times diluted and seeded in 12 well transwell inserts (Costar Corning Incorporated, Saint Louis, MO, USA). Cells were grown for 4 weeks to reach confluency and establish differentiation. Differentiation and barrier integrity of cells was assessed by trans-epithelial electrical resistance (TEER) using a Millicell ERS-2 Volt-ohm meter (Merck Millipore, Burlington, NJ, USA). IEC were exposed to 10 ng/mL IL4, IL5, IL9 or IL13 (Prospec, Ness-Ziona, Israël) to explore induction of type 2 mediated barrier disruption. HMOS were added 24 h prior to barrier disruption by 10 ng/mL IL13. The degree of barrier disruption was measured TEER in the following 48 h.

### 2.5. IEC/PBMC Coculture Model

The human colon cancer cell line HT-29 ((HTB38) ATCC, Manassas, VA, USA) was used as a model for intestinal epithelium in the IEC/PBMC coculture model. The HT-29 cells were cultured in McCoy 5A medium (Gibco, Waltham, MA, USA) supplemented with 10% FCS and the antibiotics penicillin (100 U/mL) and streptomycin (100 μg/mL) (Sigma-Aldrich, Saint Louis, MO, USA) in 75 cm^2^ cell culture flasks (Greiner Bio-One, Alphen aan den neRijn, The Netherlands). Cells were incubated at 37 °C and 5% CO_2_. Medium was refreshed every 2–3 days, and cells were cultured until 80–90%, and upon trypsinization, 5× diluted cell suspensions were seeded in 12 wells transwell inserts (Costar Corning Incorporated, Saint Louis, MO, USA). Cells were grown for 6 days to reach confluency under normal culturing conditions. After 24 h of preincubation with the 5 selected HMOS (at 0.1% and 0.5% concentrations), the cell culture medium, including HMOS, was refreshed, and IEC were basolateral exposed to freshly isolated PBMCs (2 × 10^6^ cells/mL) activated with anti-CD3 (150 ng/mL, clone CLB-T3/2) and anti-CD28 (100 ng/mL, clone CLB-C28) (Sanquin, Amsterdam, The Netherlands). IEC was exposed to apically added CpG (0.5 μM CpG oligodeoxynucleotide (ODN) M362 type C, Invivogen, San Diego, CA, USA). Basolateral supernatant was collected and stored at −20 °C until further analysis after 24 h coculturing of IEC with PBMCs. Subsequently, IEC were separated from the PBMCs and washed with PBS before transferring to a new plate. IEC were cultured for an additional 24 h in fresh medium in the absence of HMOS, and basolateral supernatants were stored at −20 °C for TGFβ and galectin-9 secretion analysis. Phenotype of PBMCs was determined with flow cytometry immediately after coculture. 

### 2.6. Enzyme-Linked Immunosorbent Assay

Stored supernatants were analyzed to quantify cytokine secretion according to the manufacturer’s protocol. Commercially available kits were used to determine IFNγ, IL9, IL13, TGFβ, TNFα (Thermo Fisher Scientific, Saint Louis, MO, USA), IL10 (U-Cytech, Utrecht, The Netherlands) and retinoic acid (MyBioSource, San Diego, CA, USA).

Galectin-9 was measured using an antibody pair (R&D Systems, Minneapolis, MN, USA). High-binding Costor 9018 plates were coated with 0.75 μg/mL affinity-purified polyclonal antibody overnight at 4 °C. After washing, non-specific binding sites were blocked with 1% BSA in PBS for 1 h before samples were incubated for 2 h at room temperature. Plates were washed before the addition of 0.75 μg/mL biotinylated galectin-9 affinity-purified polyclonal antibody. After a 1 h incubation at room temperature, plates were washed again and streptavidin-HRP (R&D Systems, Minneapolis, MN, USA) was added for 30 min. Next, tetramethylbenzidine (TMB, Thermo Fisher Scientific, Saint Louis, MO, USA) was used as a substrate, and H2SO4 was used to stop the reaction. Optical density was measured using a Promega GloMax microplate reader at 450–655 nm. 

### 2.7. Flow Cytometry Analysis

After 24 h of IEC/PBMC co-culture, PBMCs were collected and stained for analysis with flow cytometry. Viability of the cells was determined using Fixable Viability Dye 780-APC Cyanine 7 (eBioscience, Saint Louis, MO, USA). Immunophenotyping and intracellular cytokine staining was performed using antibodies with appropriate isotypes (eBioscience, Saint Louis, MO, USA; Invitrogen, Saint Louis, MO, USA) (for the list of antibodies, clones and dilutions, see Table S1). Nonspecific binding was prevented by blocking for 15 min with PBS containing 2.5% FCS and Human FC Block (BD Biosciences, Franklin Lakes, NJ, USA) before extracellular and intracellular staining. Cells were fixated and permeabilized with the FoxP3/Transcription Factor Staining Buffer Set or Intracellular Staining Buffer Set (eBioscience, Saint Louis, MO, USA). PBMC measurements was performed using BD FACS Canto II (BD Biosciences, Franklin Lakes, NJ, USA), and data were analyzed by Flowlogic software, version 8.4 (Inivai Technologies, Melbourne, Australia). 

### 2.8. Statistical Analysis

Data were analyzed using Graphpad Prism software (v8.4.0). Analysis was performed by One-Way ANOVA followed by Bonferroni multiple comparison or Dunnett post hoc test. When data did not fit a normal distribution, square root or logarithmic transformations were performed prior to analysis. Data are represented as mean ± SEM using healthy, independent PBMC donors for *n* = 3, *n* = 6 or *n* = 9 independent experimental replicates. * *p* < 0.05, ** *p* < 0.01, *** *p* < 0.001, **** *p* < 0.0001. 

## 3. Results

### 3.1. Direct Immunomodulatory Effects of Specific HMOS, Leading to Th1 Type and Regulatory Cytokine Responses from Activated PBMCs

**Figure 2:**
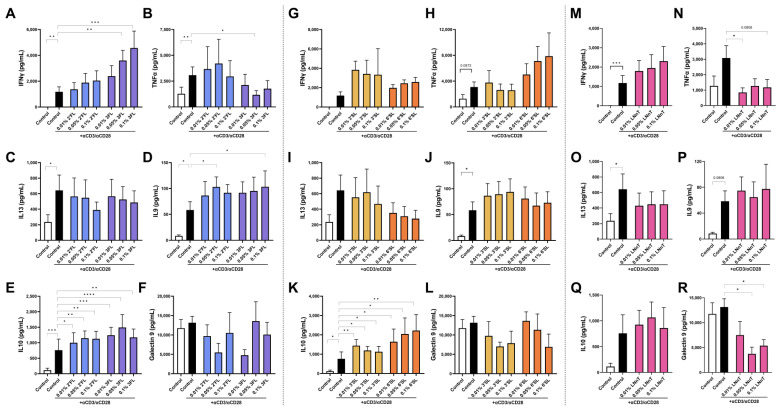
PBMCs were exposed directly low concentrations of all HMOS for 24 h while activated using αCD3 and αCD28. Concentrations of (**A**,**G**,**M**) IFNγ, (**B**,**H**,**N**) TNFα, (**C**,**I**,**O**) IL13, (**D**,**J**,**P**) IL9, (**E**,**K**,**Q**) IL10 and (**F**,**L**,**R**) galectin-9 were assessed in the supernatant. Data is analyzed by One-Way ANOVA followed by a Dunnett post-hoc test, *n* = 6 independent experiments using different donors, mean ± SEM (* *p* < 0.05, ** *p* < 0.01, *** *p* < 0.001, **** *p* < 0.0001).

First, the direct immunomodulatory effects of the HMOS on freshly isolated human PBMCs were studied, as HMOS are able to cross intestinal epithelial cells to interact with receptors on immune cells [30]. The effects of exposure to low concentrations (0.01%, 0.05% and 0.1%) of HMOS on non-activated PBMCs are shown in Table 2. After exposure to HMOS, PBMCs secreted in general higher levels of IFNγ, which was significantly enhanced with 0.1% 3FL exposure (Table 2). Furthermore, an increase in IL10 secretion was observed after 0.1% 6′SL exposure. The HMOS 2′FL, 3FL, 3′SL and LNnT reduced the levels of galectin-9 in PBMC supernatant compared to the medium control while 6′SL did not. Upon activation using anti-CD3 and -CD28, the PBMCs increased the secretion of IFNγ, TNFα, IL13, IL9 and IL10 (Figure 2). The activated PBMCs were exposed to HMOS for 24 h, mimicking systemic concentrations [7]. An analysis of secreted cytokines revealed that exposing activated PBMCs to 2′FL enhanced the secretion of IL9 and IL10 (Figure 2D,E) while the secretion of other cytokines was hardly affected compared to the activated control. In addition, 3FL exposure resulted in an increase in IL10 (Figure 2E) as well, and a dose dependent increased the secretion of IFNγ (Figure 2A) while TNFα levels were decreased (Figure 2B). Both sialylated HMOS, 3′SL and 6′SL, induced an increase in IL10 secretion from PBMCs, and even though 3′SL showed an inclining pattern for IFNγ and IL9 and 6′SL for TNFα, this did not reach significance (Figure 2K). in contrast, the undecorated LNnT only reduced the secretion of proinflammatory TNFα and basal levels of galectin-9 (Figure 2N,R). 

### 3.2. IL13 Induced Barrier Disruption in T84 Cells Is Not Prevented by HMOS Pre-Exposure

**Figure 3:**
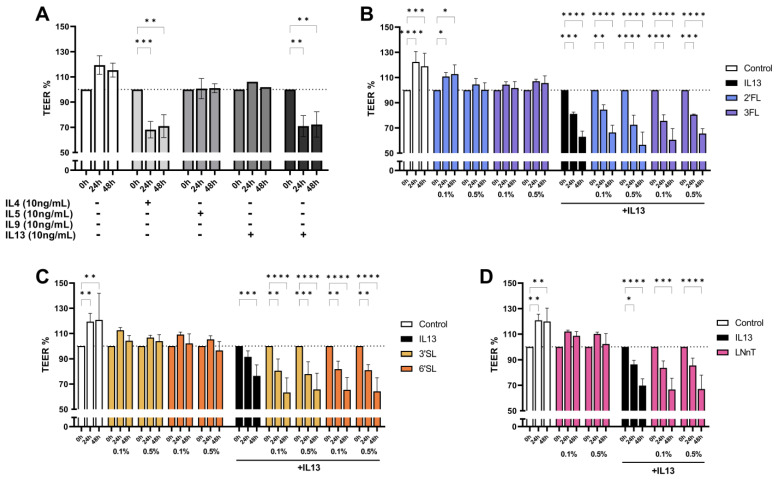
Effects of HMOS on IL13-mediated IEC barrier disruption. T84 cells were grown on transwells and (**A**) exposed to the Th2-type cytokines IL4, IL5, IL9 and IL13 after which transepithelial electrical resistance (TEER) was measured at 24 h and 48 h. To investigate preventive effects of HMOS on barrier disruption by IL13, IEC were preincubated with (**B**) 2′FL and 3FL, (**C**) 3′SL and 6′SL, and (**D**) LNnT 24 h prior to exposure to IL13. Data is analyzed by Two-Way ANOVA followed by a Bonferroni post-hoc test, *n* = 6, mean ± SEM (* *p* < 0.05, ** *p* < 0.01, *** *p* < 0.001, **** *p* < 0.0001).

Knowing the important role of the epithelial barrier in tolerance induction and strengthening of Th1 immunity to compensate for Th2 skewing in early life, the differential effects of type 2 inflammatory mediators on barrier disruption using T84 cells was studied. Confluent and differentiated T84 cells were basolateral exposed to the type 2 related cytokines IL4, IL5, IL9 and IL13 for 48 h. Barrier integrity, determined by TEER, was decreased upon exposure to IL4 and IL13, yet IL5 and IL9 did not affect the barrier integrity (Figure 3A). As IL4 and IL13 are known to bind similar receptors (e.g., type II IL4Rα) [31], further experiments were conducted using only IL13. Subsequently, confluent and differentiated T84 cells were apically pre-exposed to 0.1% or 0.5% 2′FL, 3FL, 3′SL, 6′SL or LNnT for 24 h. HMOS were refreshed while IL13 was added to the basolateral compartment for 48 h. The development of TEER values was followed after IL13 exposure. As depicted in Figure 3B–D, none of the HMOS affected basic barrier properties of the T84 cells in the absence of IL13 nor prevented the IL13 induced barrier disruption.

### 3.3. Crosstalk between IEC and PBMC Is Differentially Affected by 2′FL and 3FL

To further investigate the immunomodulatory effects of the HMOS 2′FL and 3FL, the well-studied HT29-activated PBMC coculture model was used [27,28,32]. Two sources (enzymatic modified lactose or bacterially produced) of 2′FL and 3FL were studied. IEC were apically exposed to the HMOS for 24 h before the apical addition of CpG and basolateral addition of anti-CD3/CD28 activated PBMC to allow cellular crosstalk. In this coculture model, it was previously shown that only under CpG-activated conditions epithelial cells were able to enhance galectin-9 expression and secretion. This correlated with increased IFNγ and IL10 secretion of underlying PBMC, which was further increased by non-digestible oligosaccharides [27,33]. 

**Figure 4:**
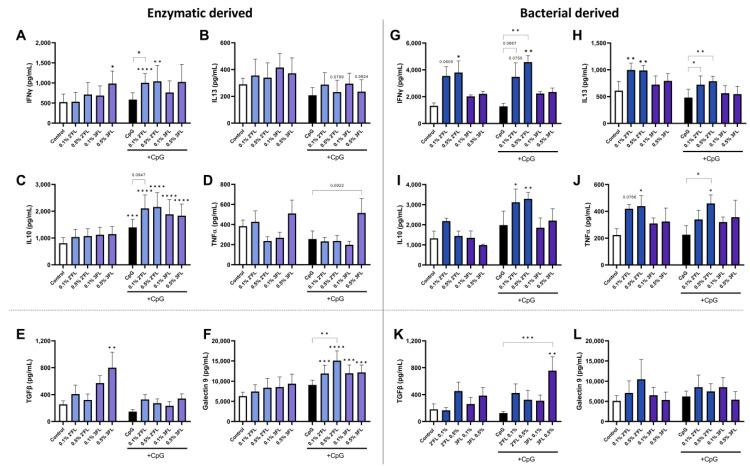
24 h after preincubation of IEC with HMOS, IEC were cocultured with activated PBMCs for another 24 h, while fresh HMOS and/or CpG were added to the apical compartment of the transwell. After this coculture, cytokine secretion was measured in the basolateral compartment. Inserts containing IEC were transferred to a new plate, HMOS and CpG were washed away and IEC were cultured for another 24 h to detect epithelial derived TGFβ and galectin-9 in the basolateral compartment. Release of (**A**) IFNγ, (**B**) IL13, (**C**) IL10 and (**D**) TNFα upon coculture of IEC and activated PBMC while exposing IEC to enzymatic derived 2′FL and 3FL, as well as IEC derived (**E**) TGFβ and (**F**) galectin-9 after coculture. In addition, release of (**G**) IFNγ, (**H**) IL13, (**I**) IL10 and (**J**) TNFα upon coculture of IEC and activated PBMC while exposing IEC to bacterial derived 2′FL and 3FL, as well as IEC derived (**K**) TGFβ and (**L**) galectin-9 IEC after coculture. Data is analyzed by One-Way ANOVA followed by a Bonferroni post-hoc test, *n* = 9 for synthetically produced HMOS, *n* = 3 for bacterial produced HMOS, mean ± SEM (* *p* < 0.05, ** *p* < 0.01, *** *p* < 0.001, **** *p* < 0.0001).

In the absence of CpG, the enzymatic 2′FL exposure did not result in altered cytokine secretion. However, in the presence of CpG, 2′FL enhanced IFNγ and IL10 secretion in the IEC/PBMC coculture, as well as IEC-derived galectin-9 from after the coculture as compared to exposure to medium and/or CpG alone (Figure 4A,C,F). Exposure to 0.1% 2′FL further increased the secretion of IFNγ and IL10 compared to the CpG control while 0.5% 2′FL further enhanced galectin-9 secretion compared to the CpG control. CpG alone only slightly enhanced IL10 secretion compared to the medium controls (Figure 4C). On the other hand, 0.5% 3FL enhanced IFNγ secretion from the IEC/PBMC coculture and TGFβ release by IEC derived from the coculture in the absence of CpG (Figure 4A,E). When combined with CpG, both concentrations of the 3FL enhanced secretion of IL10 from IEC/PBMC coculture and galectin-9 from IEC compared to the medium controls (Figure 4C,F). 

When the coculture was performed with bacteriall**y** derived 2′FL and 3FL, different cytokine secretion profiles were found. Both concentrations of 2′FL enhanced IFNγ and IL13, both in the presence or absence of CpG compared to the medium or CpG alone (Figure 4G,H). In addition, 0.5% 2′FL also significantly enhanced TNFα levels independent of CpG exposure and IL10 in the presence of CpG (Figure 4I,J). Bacterially derived 3FL did not show any immunomodulatory effects but increased TGFβ secretion from IEC at 0.5% only when combined with CpG (Figure 4K). 

These data indicate that 2′FL and 3FL differentially enhance Th1 type, Th2 type and regulatory cytokine secretion from IEC and PBMC coculture depending on the 2′FL and 3FL production method. Therefore, the production method affects the immunomodulatory properties of HMOS and should be taken into account when designing experiments and interpreting their outcomes. As the most profound direct immunomodulatory effects were found for 2′FL and 3FL, this manuscript will further focus on these HMOS structures. The findings for 3′SL, 6′SL and LNnT are presented in Figure S1. 

### 3.4. Enzymatic 3FL Decreases Th2 Type Development and Enhances the Polarization of Tregs

**Figure 5:**
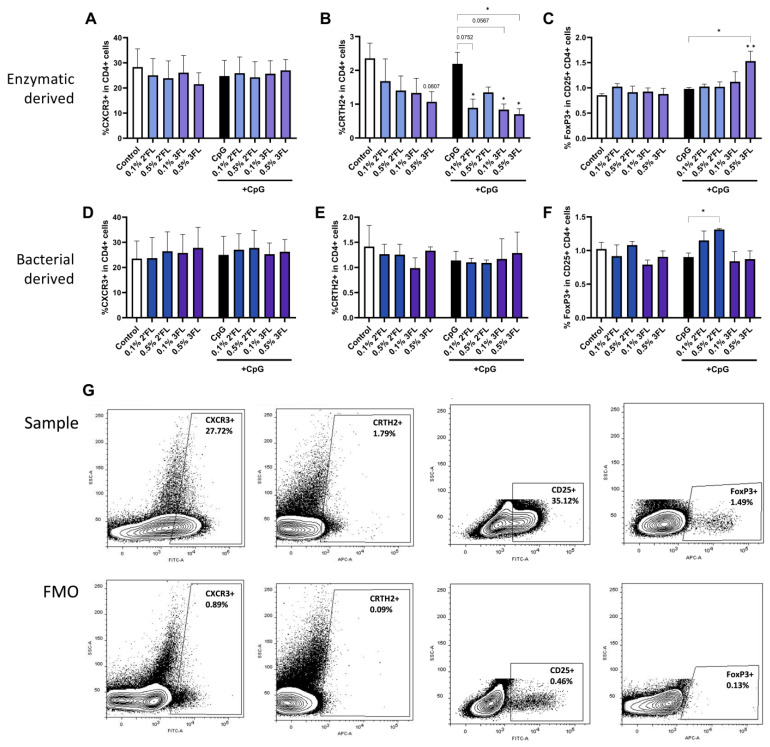
After 24 h coculture of HMOS and CpG exposed IEC cocultured with activated PBMCs, basolateral PBMCs were collected for phenotypical analysis by flow cytometry. Expression (**A**,**D**) of CXCR3, as well as (**B**,**E**) CRTH2 in CD4+ cells was assessed to represent Th1-type and Th2-type cells. Expression of (**C**,**F**) FoxP3 + in CD25 + CD4 + cells was assessed, representing Treg cells. The upper row displays results of enzymatic derived 2′FL and 3FL, while the lower row displays bacterial derived 2′FL and 3FL. (**G**) A representative sample and corresponding FMO controls displaying the gating strategy of Th cell subset markers. Data is analyzed by One-Way ANOVA followed by a Bonferroni post-hoc test, *n* = 3, mean ± SEM (* *p* < 0.05, ** *p* < 0.01).

After 24 h of IEC/PBMC coculture, PBMCs were collected for flow cytometric analysis. Percentages of Th1 type (CXCR3 + in CD4 + cells), Th2 type (CRTH2 + in CD4 + cells) and Tregs (FoxP3 + in CD25 + CD4 + cells) were determined. A representative example of flow cytometry gating is shown in Figure 5G. The percentage of Th1 type cells was not affected by exposure to 2′FL or 3FL irrespective of the presence of CpG (Figure 5A,D). However, the percentage of Th2 type cells was decreased by enzymatically derived 0.1% 2′FL and both concentrations 3FL, but not by their bacterial analogues, compared to CpG exposed cells (Figure 5B,E). In addition, bacterially derived 0.5% 2′FL enhanced the frequency of the Treg population (Figure 5F), which was not observed for the enzymatically derived 2′FL (Figure 5C). Furthermore, only the enzymatically derived 0.5% 3FL increased the percentage of Treg cells (Figure 5C). 

### 3.5. IEC Are Required for 3FL Mediated Treg Polarization and IEC Enhance 2′FL and 3FL Mediated Th1 Type and Th2 Type Cytokine Responses

Epithelial-derived regulatory mediators such as galectin-9, TGFβ and retinoic acid are known to contribute to the differentiation of Tregs [34,35,36]. Therefore, the role of IEC in the immunomodulatory effect of enzymatic 2′FL and 3FL was investigated by comparing HMOS exposure in the absence and presence of HT29 cells in the IEC/PBMC coculture model. 

**Figure 6:**
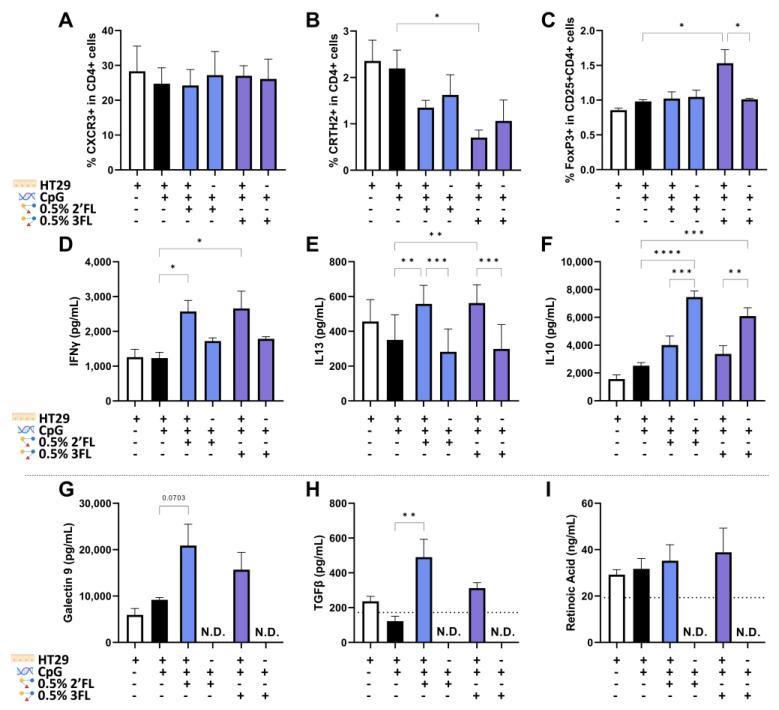
The role of IEC in HMOS plus CpG mediated immunomodulation. This was assessed by comparing PBMC that were exposed to 2′FL and 3FL combined with CpG in absence or presence of IEC in the transwell. Th1-type, Th2-type and Treg cells were assessed by expressing (**A**) CXCR3 in CD4 + cells, (**B**) CRTH2 in CD4+ cells and (**C**) FoxP3 in CD25 + CD4+ cells. In addition, secretion of (**D**) IFNγ, (**E**) IL13, and (**F**) IL10 in the basolateral compartment was determined after exposure to 2′FL and 3FL combined with CpG in absence or presence of IEC. In addition, epithelial-derived (**G**) galectin-9, (**H**) TGFβ and (**I**) retinoic acid were measured when IEC were set apart after coculture. Basal concentrations in culture medium are indicated with the dotted line, N.D. is mentioned when concentrations were not determined. Data is analyzed by One-Way ANOVA followed by a Bonferroni post-hoc test, *n* = 3, mean ± SEM (* *p* < 0.05, ** *p* < 0.01, *** *p* < 0.001, **** *p* < 0.0001).

The differentiation of Th1 (CXCR3 + CD4 cells) and Th2 (CRTH2 + CD4) cells was not significantly affected during exposure to either 2′FL or 3FL combined with CpG in the absence of IEC compared to the presence of IEC (Figure 6A,B). However, the improved Treg development upon the exposure of IEC to CpG and 3FL was abrogated when IEC were not present (Figure 6C). The combined exposure to CpG and 2′FL or 3FL enhanced the secretion of IFNγ and IL13 in the presence of IEC compared to exposure to CpG alone. This effect was lost when IEC were not present (Figure 6D,E). On the other hand, the secretion of IL10 in response to CpG plus 2′FL or 3FL was significantly further enhanced when IEC were not present (Figure 6F). After the coculture, IEC were washed and set apart for another 24 h to measure epithelial-derived regulatory mediators. Galectin-9 and TGFβ concentrations were significantly increased upon the exposure of IEC to 2′FL, and 3FL showed a similar pattern compared to control IEC exposed to CpG alone (Figure 6G,H). Retinoic acid produced by the IEC was also detected, but no significant differences were found in the used conditions (Figure 6I). The epithelial-derived mediators in Figure 6G–I are not displayed in the conditions without IEC; the medium background levels are indicated with a dotted line. 

## 4. Discussion

HMOS are thought to play a pivotal role in the development of the gut microbiome, maturation of the gastrointestinal tract and shaping of a resilient and innate and adaptive immunity in infants [30]. The relative high abundance of fucosylated and sialylated oligosaccharides in human milk especially is unique among mammals [37]. HMOS promote the intestinal barrier integrity and support innate and adaptive immune responses [30,38,39,40]. Aside from acting on the immune system via the microbiome, HMOS have also been found to directly interact with immune cells [41]. These direct effects are difficult to distinguish in in vivo models and clinical studies due to the presence of the microbiome. Furthermore, the large structural variety of HMOS makes identifying structure–function relationships from pooled HMOS challenging, although improved immune maturation has been attributed to the total mixture of HMOS present in human milk [41,42,43,44]. Using an established transwell mucosal immune model combining both IEC and activated PBMC coculture and the presence of a bacterial or allergic trigger, differential immunomodulatory properties of specific HMOS have been identified. The current study was designed to explore the immunomodulatory effects of 5 different HMOS: 2′FL, 3FL, 3′SL, 6′SL and LNnT, which are currently produced in larger quantities.

Antigen exposure to the mucosal immune system may lead to (mucosal) immune activation, as may be the case in early infancy due to immature gut barrier function. This further drives immune maturation and tolerance development, or alternatively may prime for developing immune disorders like food allergy in the case of a type 2 prone immune polarization [45]. To study whether the HMOS are able to modify the direction of immune activation, PBMCs were exposed to a low dosage of HMOS, as it is known that a small fraction of the ingested HMOS can become systemically available after ingestion [7,46,47]. Only limited effects on cytokine secretion from non-activated PBMCs were observed after HMOS exposure (Table 2). Therefore, PBMCs were activated using anti-CD3 and -CD28 antibodies, which initiate inflammatory and regulatory cytokine release via activating the T cells within the PBMCs. Both fucosylated and sialylated HMOS significantly enhanced the secretion of the regulatory cytokine IL10 in activated PBMCs, which was not observed in non-activated PBMCs. Previously, it was shown that monocyte-derived dendritic cells exposed to pooled HMOS enhance IL10 secretion [41]. However, enhanced IL10 secretion for the undecorated LNnT was not observed, which did reduce the release of proinflammatory TNFα. Furthermore, 3FL, but not the structurally similar 2′FL, enhanced secretion of type 1 IFNγ while also reducing TNFα release, potentially promoting a regulatory Th1 response important for immune maturation. These fucosylated HMOS are structurally similar and comparable immunomodulatory effects could be hypothesized. However, these present data demonstrate that the structurally similar 2′FL and 3FL interact differently with PBMCs, which is in line with differences in receptor affinity profiles described for these fucosylated HMOS as previously reviewed [30]. 

Although the high abundance of fucosylated oligosaccharides distinguishes human milk from milk from other mammalian species, sialylated oligosaccharides are present in relatively high levels in the milk of most mammals [48]. Sialylated HMOS have been linked to cognitive development and health via the direct supply of sialic acid to the brain or indirectly via the gut–brain axis [49]. Therefore, investigating their immunomodulatory properties is relevant to gain insights into potential mechanistic routes. Although a few studies have looked into the effects of 3′SL and 6′SL on epithelial cells [50,51], the direct immunomodulatory effects have been questioned [52,53]. This current study demonstrates an increase in IL10 release while proinflammatory cytokines were unaffected by 3′SL and 6′SL in activated PBMCs. These effects were only observed after the activation of PBMCs; in non-activated PBMCs galectin-9 levels dropped with 3′SL exposure and only 0.1% 6′SL enhanced IL10 secretion. Combined with the relatively limited effects in the IEC/PBMC mucosal coculture model (Figure S1), these data indeed indicate minimal immunomodulatory effects, in the currently used models, mediated by these specific sialylated HMOS. Nevertheless, a role within the complex mixture of HMOS or other cell models cannot be excluded. 

The final HMOS structure investigated in this study is LNnT, a neutral, less abundant and undecorated HMOS. LNnT has been studied mainly in preclinical and clinical settings; however, in vitro findings show that LNnT has affinity for galectins, including galectin-9, which has a regulatory function [21,25,54]. Here, LNnT lowered TNFα and galectin-9 when added directly to activated PBMC; however, these effects were lost when immunomodulatory effects of LNnT were studied in the mucosal immune coculture of IEC/PBMC (Figure S1). Preclinical and clinical studies demonstrate positive effects from LNnT supplementation on microbiota composition [55], which is supported by only a few in vitro studies reporting improved epithelial maturation and proliferation during exposure to LNnT [50,56]. However, to the best of our knowledge, this is one of the first studies investigating direct effects of LNnT on immune cells. 

HMOS support the intestinal epithelial barrier of Caco-2:HT29-MTX cultures under healthy and inflammatory conditions [57] and may, therefore, support gut maturation in early infancy as well. For example, 2′FL reduces the secretion of proinflammatory cytokines while enhancing epithelial barrier integrity during LPS or chemotherapy exposure in vitro [57,58,59], yet 3FL increased the expression of MUC2, encoding the most abundant protein in intestinal mucus, in LS174T cells during TNFα and IL13 exposure [60]. In the present study, we could not detect effects on the epithelial tight junction barrier integrity with allergic phenotype (i.e., challenged with IL13), nor in the absence of an inflammatory trigger. However, studies investigating HMOS and barrier integrity mainly focus on fermentation products of HMOS rather than the direct effects of HMOS themselves, nor include a type 2 inflammatory milieu, which is relevant during early life [61,62]. The current study does not indicate a direct effect of the diverse HMOS structures on epithelial integrity, and no protection against type 2 related barrier disruption was observed. 

Beyond providing a barrier, intestinal epithelial cells however actively contribute to shaping of mucosal immune function as well. Therefore, we investigated the effects of HMOS on the immunological crosstalk between human IEC and immune cells using a validated HT-29/PBMC coculture model, which was developed for this purpose [27,28,63,64]. In this model the two individual sialylated HMOS or LNnT did not give a clear immunomodulatory signature (Figure S1); therefore, next steps were focused on 2′FL and 3FL. 

Recently, techniques have been developed to produce HMOS on larger scale, which makes these structures available for commercial purposes. Enzymatically and bacterially produced 2′FL and 3FL have been compared and it was demonstrated that the most pronounced immunomodulatory effects with these HMOS were shown on activated PBMCs. CpG is added in this model as an analogue for bacterial DNA (binding to TLR9) driving a regulatory type 1 response, which is amplified by several oligosaccharides as was previously investigated [27,28,32,33]. In this manuscript, immunological responses were found to differ between enzymatic and bacterial produced HMOS. These differences could not be ascribed to differences in endotoxin contaminations in enzymatic or bacterial produced HMOS (presented in Table 1). The endotoxin contamination level of all used HMOS is low and not in a range to expect to significantly contribute to the immunological responses [33,65]. In the presence of CpG, enzymatic 2′FL further enhanced regulatory and Th1 type cytokine release from PBMCs and galectin-9 secretion from IEC, while also diminishing Th2 cell development. Bacterial 2′FL enhanced the cytokine release in general while enhancing Treg development, in the presence of CpG. In the absence of CpG bacterial 2′FL enhanced the secretion of both type 1 and type 2 related cytokines including pro-inflammatory TNFα, while regulatory cytokine secretion and Th cell differentiation was unaffected. Bacterial derived 3FL did not have these immune stimulatory effects, but was found to enhance epithelial derived TGFβ secretion in the presence of CpG. 

Similar to enzymatic produced 2′FL also enzymatic produced 3FL, enhanced IL10 in the IEC/PBMC coculture in the presence of CpG while increasing galectin-9 release by the epithelial cells. Even though 3FL did not further enhance IFNγ in the CpG conditions, 0.5% 3FL significantly enhanced Treg cell development combined with a reduction in Th2 cells. These data indicate that bacterial derived 2′FL generally drives immune activation while 3FL is less effective. By contrast, enzymatic 2′FL and 3FL selectively drive away from the allergic phenotype while enhancing either a regulatory type Th1 (2′FL) or regulatory T cell response (3FL) in association with increase epithelial derived galectin-9 secretion. Previous studies have indicated epithelial derived galectin-9 to correlate positively with IFNγ and/or IL10 release by the activated PBMC [27,28], which may be one of the mechanisms by which 2′FL and 3FL exposed IEC are able to modify mucosal immune function and influence immune maturation. Thus, these results demonstrate different immunomodulatory outcomes from the structurally similar 2′FL and 3FL. Furthermore, the bacterial versus enzymatic origin of HMOS plays a role in immunological outcomes and should therefore be taken into account in future studies when investigating HMOS. To our knowledge this is the first study comparing manufactured HMOS from different origins. Future experiments should also include biological HMOS isolated from human milk as an internal standard for immunomodulation. 

To further investigate the role of IEC in the immunomodulatory effects of 2′FL and 3FL combined with CpG, the IEC/PBMC coculture experiments were performed in the presence or absence of IEC in the transwell using only the enzymatic produced 2′FL and 3FL. Previously the type 1 supporting effects of nondigestible oligosaccharides was shown to depend on the presence of IEC in the CpG exposed IEC/PBMC coculture model [27]. Indeed also in the current study, in the presence of CpG, 2′FL was found to enhance type 1 IFNγ release only in the presence of IEC. CpG can contribute to reduced type 2 IL13 secretion in the HT-29/PBMC coculture model [27,63] which may be further enhanced by nondigestible oligosaccharides. This effect was not shown for 2′FL and 3FL, however it appeared that direct exposure of activated PBMC to CpG in absence of IEC resulted in a reduced type 2 response. CpG is a bacterial DNA surrogate known for its capacities to drive away from the allergic phenotype and immunoregulatory properties [33]. This may also explain the further increase in regulatory type IL10 secretion when 2′FL and 3FL plus CpG were exposed to activated PBMC in absence of IEC. 

In particular, 3FL was found to decrease the Th2 type population while increasing the Treg population. Interestingly, this effect was depended on the presence of IEC, which was most prominent for Treg development. Mediators produced by epithelial cells in this coculture model may be required for the impact on polarization patterns in Th cells as was previously shown for epithelial derived galectin-9. Galectin-9 was found to contribute to increased IFNγ, IL10 secretion and Treg development in activated PBMC [27,52]. Therefore, we measured the epithelial-derived regulatory mediator galectin-9. Furthermore, TGFβ and retinoic acid are known to be able to induce Treg formation [34,35,36]. Galectin-9 was indeed secreted by the epithelial cells, yet not significantly increased by 2′FL and 3FL on top of the CpG. However, as indicated galectin-9 is known to contribute to type 1 and regulatory type immune responses and may also play a role in the immunomodulatory effects of 2′FL and 3FL. In addition, in the 2′FL exposed conditions epithelial derived TGFβ was increased, which indicates that 2′FL indeed is able to enhance the regulatory function of epithelial cells. However, the increase in Treg population supported by 3FL in the presence of IEC and CpG could not be explained by enhanced release of TGFβ and/or retinoic acid by the epithelial cells. Future experiments should therefore focus on additional factors derived from IEC contributing to Treg development in underlying immune cells. 

To validate the findings in the current manuscript, more complex mucosal immune models that mimic the sequential steps in mucosal immune activation should be used to further investigate the immunomodulatory effects of different HMOS. Furthermore, this study only investigated five relatively simple HMOS structures while the total pool of HMOS in human milk contains many more different structures. This leaves opportunities to investigate other single structures as well as mixtures of HMOS which could potentially lead to an optimized HMOS mixture suitable to be added to infant formula in order to promote the optimal immune development of infants that cannot receive breast milk

## 5. Conclusions

HMOS are thought to possess immunomodulatory effects; however, it has only recently become possible to study specific HMOS structures that can be produced for commercial purposes. In this current the effects of five HMOS; 2′FL, 3FL, 3′SL, 6′SL and LNnT in several in vitro models for intestinal barrier and systemic or mucosal immune modulation have been investigated. Although none of the HMOS protected against type 2 mediated barrier disruption, these data demonstrate the most pronounced immunomodulatory effects for 2′FL and 3FL. This was affected by the HMOS origin and the presence or absence of IEC combined with CpG. 3′SL, 6′SL and LNnT had minimal immunomodulatory effects in the models tested. Enzymatically produced 3FL showed a regulatory type Th1 immune supportive effect while lowering general inflammation when added directly to activated PBMC. Both enzymatically produced 2′FL and 3FL showed regulatory effects when combined with CpG in the mucosal IEC/PBMC immune model, but in this model 2′FL also increase type Th1 immunity while 3FL enhanced Treg conversion. Future studies using more complex mucosal immune models and/or using more specific immune cell types are warranted to further study the direct immunomodulatory capacities of complete collections of HMOS in order to further understand their role in early life immune development and their potential application in infant formula.

## Figures and Tables

**Table 1:** Origin, purity and endotoxin content of the HMOS used in the experiments.

Structure	Origin	Purity	Endotoxin (EU/mg)
2′FL	Enzymatic	>95%	0.03
3FL	Enzymatic	96.8%	0.0626
2′FL	Bacterial	92.7%	0.0175
3FL	Bacterial	94.1%	0.025
LNnT	Enzymatic	99.9%	0.0616
3′SL	Enzymatic	95.8%	0.0516
6′SL	Enzymatic	98.1%	1.023

**Table 2:** Effects of HMOS on mediator secretion from nonactivated PBMCs.

(pg/mL)	Control	0.01% 2′FL	0.05% 2′FL	0.1% 2′FL	0.01% 3FL	0.05% 3FL	0.1% 3FL	0.01% 3′SL	0.05% 3′SL	0.1% 3′SL	0.01% 6′SL	0.05% 6′SL	0.1% 6′SL	0.01% LNnT	0.05% LNnT	0.1% LNnT
**IFNγ**	5.7 ± 3.5	18.5 ± 11.8	41.3 ± 23.6	145.7 ± 108.3	40.7 ± 35.4	182.5 ± 87.9 ^1^	193.5 ± 101.8 *	77.2 ± 36.2	125.4 ± 45.7	63.5 ± 23.5	5.7 ± 3.6	144.2 ± 65.6	78.2 ± 37.8	19.8 ± 12.1	38.3 ± 15.3	164.6 ± 94.6
**TNFα**	1424 ± 802.8	1195 ± 746.0	1185 ± 476.4	703.8 ± 258.2	633.6 ± 260.2	699.4 ± 142.6	905.1 ± 257.6	1083 ± 455.4	826.5 ± 332.6	673.8 ± 248.5	2526 ± 1922	2175 ± 1129	2757 ± 1501	700.5 ± 282.1	908.7 ± 499.5	980.5 ± 528.1
**IL13**	236.4 ± 91.4	128.2 ± 39.6	123.8 ± 50.5	167.7 ± 71.1	128.2 ± 56.4	181.6 ± 55.5	184.0 ± 57.0	101.0 ± 57.0	160.2 ± 93.4	82.9 ± 32.1	253.3 ± 100.4	129.4 ± 37.5	119.3 ± 24.4	158.1 ± 68.	105.5 ± 33.6	139.3 ± 35.8
**IL9**	8.5 ± 1.9	17.8 ± 7.0	33.2 ± 16.9 ^2^	22.8 ± 4.7	23.9 ± 8.7	16.2 ± 4.7	16.3 ± 6.4	20.8 ± 6.3	24.8 ± 7.3	17.1 ± 5.2	15.4 ± 3.5	20.3 ± 4.1	26.2 ± 9.9	27.2 ± 7.2	21.4 ± 7.2	18.9 ± 6.5
**IL10**	113.9 ± 63.7	134.4 ± 61.8	164.4 ± 92.0	398.7 ± 160.8	68.4 ± 46.2	164.1 ± 136.0	161.5 ± 90.4	296.4 ± 163.5	269.5 ± 142.6	250.2 ± 144.1	111.5 ± 41.0	442.7 ± 139.6	612.9 ± 127.4 *	156.3 ± 53.5	58.1 ± 36.6	153.6 ± 111.8
**Gal-9**	11755 ± 2229	5386 ± 1669 *	3646 ± 1693 **	4740 ± 1435 *	2221 ± 655.1 ***	3041 ± 1131 **	2777 ± 924.6 ***	1483 ± 332.8 ***	1815 ± 575.6 ***	4325 ± 2077 *	9040 ± 1097	9219 ± 3542	4470 ± 1395 *	2604 ± 1087 ***	3828 ± 1111 ***	3122± 1090 ***

All conditions were compared to Control using a One-Way ANOVA with Dunnett post-hoc test (*n* = 6, ^1^ *p* = 0.0740, ^2^ *p* = 0.0813, * *p* < 0.05, ** *p* < 0.01, *** *p* < 0.001).

## Data Availability

The data presented in this study are available on request from the corresponding author.

## Supplementary Materials

The following supporting information can be downloaded at: https://www.mdpi.com/article/10.3390/biom13020263/s1, Figure S1: Immunomodulatory effects of 3′SL, 6′SL and LNnT in the IEC/PBMC coculture; Table S1: Overview of flow cytometry antibodies
