# Supplementary material for: Specific Human Milk Oligosaccharides Differentially Promote Th1 and Regulatory Responses in a CpG-Activated Epithelial/Immune Cell Coculture
Source: Biomolecules. 2023 Jan 31;13(2):263. doi: 10.3390/biom13020263 (PMC9953370; doi:10.3390/biom13020263)
Supplement: Supplementary file 1 [file biomolecules-13-00263-s001.zip › biomolecules-2149735-supplementary.pdf]

Supplemental Material

Table S1 Overview of antibodies used for flow cytometry. Appropriate isotype controls were used for FMO stainings. All antibodies were purchased from eBioscience or Biolegend and titrated prior to use.

**Marker**	**Fluorochrome**	**Clone**	**isotype**	**Dilution**
CD4	PerCP Cyanine 5.5	OKT4	m IgG2b, k	1:320
CD25	Alexa Fluor 488	BC96	m IgG1, k	1:150
CXCR3	Alexa Fluor 488	1C6/CXCR3	m IgG1, k	1:80
CRTH2	APC	BM16	r IgG2a, k	1:150
FoxP3	APC	PCH101	r IgG2a, k	1:320

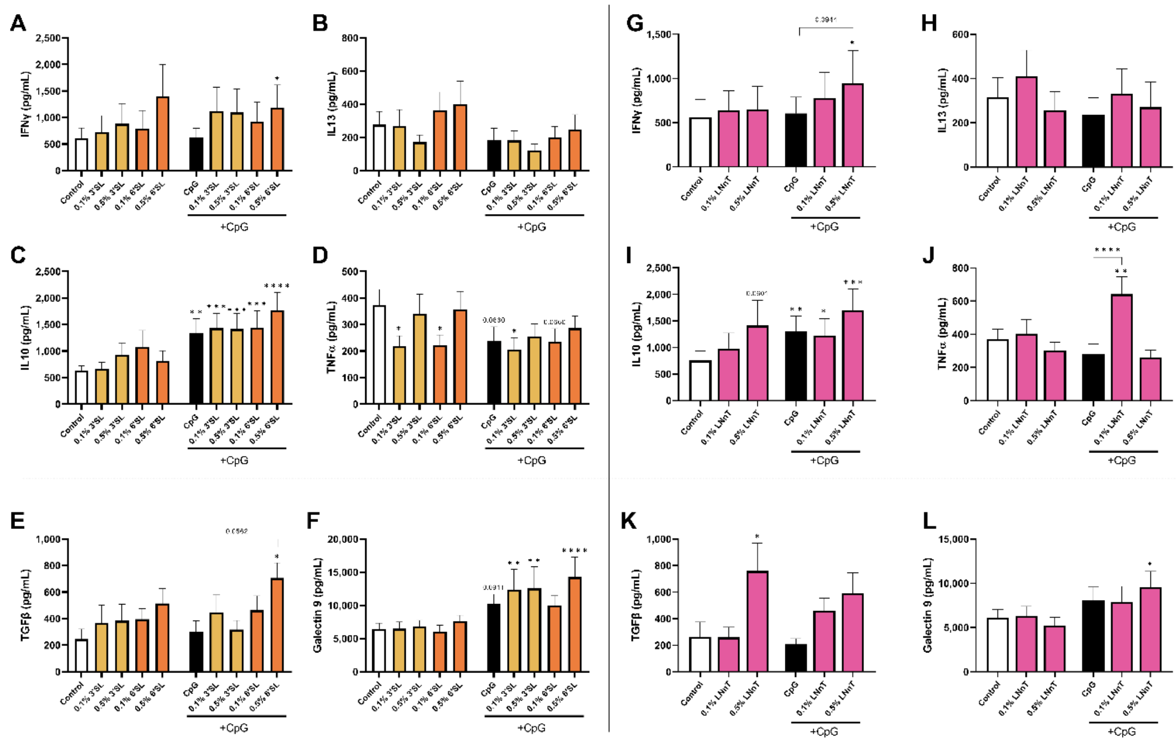

Figure S1. 24h After preincubation of IEC with HMOS, IEC were cocultured with activated PBMCs for another 24h while fresh HMOS and/ or CpG were added to the apical compartment of the transwell. After this coculture, cytokine secretion was measured in the basolateral compartment. Inserts containing IEC were transferred to a new plate, HMOS and CpG were washed away and IEC were cultured for another 24h to detect secreted TGFβ and galectin-9 in the basolateral compartment. Release of A) IFNγ, B) IL13, C) IL10 and D) TNFα upon coculture of IEC and activated PBMC with synthetic produced 3′SL and 6′SL, as well as IEC derived E) TGFβ and F) galectin-9 after coculture. In addition, release of G) IFNγ, H) IL13, I) IL10 and J) TNFα upon coculture of IEC and activated PBMC while exposing IEC to synthetic produced LNnT was determined, as well as IEC derived K) TGFβ and L) galectin-9 after coculture. Data is analyzed by One-Way ANOVA followed by a Bonferroni post-hoc test, *n* = 9 independent experiments using different PBMC donors, mean ± SEM (* p<0.05, ** p<0.01, *** p<0.001, ****p<0.0001).
